# ETS1 and HLHS: Implications for the Role of the Endocardium

**DOI:** 10.3390/jcdd9070219

**Published:** 2022-07-08

**Authors:** Paul Grossfeld

**Affiliations:** Department of Pediatrics, Division of Cardiology, UCSD School of Medicine, San Diego, CA 92093, USA; pgrossfeld@health.ucsd.edu

**Keywords:** hypoplastic left heart syndrome, endocardium, Jacobsen syndrome, cardiac myocyte, hyperplasia

## Abstract

We have identified the ETS1 gene as the cause of congenital heart defects, including an unprecedented high frequency of HLHS, in the chromosomal disorder Jacobsen syndrome. Studies in *Ciona intestinalis* demonstrated a critical role for ETS1 in heart cell fate determination and cell migration, suggesting that the impairment of one or both processes can underlie the pathogenesis of HLHS. Our studies determined that ETS1 is expressed in the cardiac neural crest and endocardium in the developing murine heart, implicating one or both lineages in the development of HLHS. Studies in *Drosophila* and *Xenopus* demonstrated a critical role for ETS1 in regulating cardiac cell fate determination, and results in *Xenopus* provided further evidence for the role of the endocardium in the evolution of the “hypoplastic” HLHS LV. Paradoxically, these studies suggest that the loss of ETS1 may cause a cell fate switch resulting in the loss of endocardial cells and a relative abundance of cardiac myocytes. These studies implicate an “HLHS transcriptional network” of genes conserved across species that are essential for early heart development. Finally, the evidence suggests that in a subset of HLHS patients, the HLHS LV cardiac myocytes are, intrinsically, developmentally and functionally normal, which has important implications for potential future therapies.

## 1. Introduction

Although there is overwhelming evidence for a genetic component underlying HLHS, surprisingly few individual genes have been identified that, together, only account for a small subset of patients [1]. This likely reflects the multifactorial etiologies of HLHS. Nonetheless, the small number of genes identified may lead to insights that are relevant to the pathogenesis of the disease in a larger subset of patients, and potentially towards the identification of additional genes involved in common regulatory networks.

**Figure 1:**
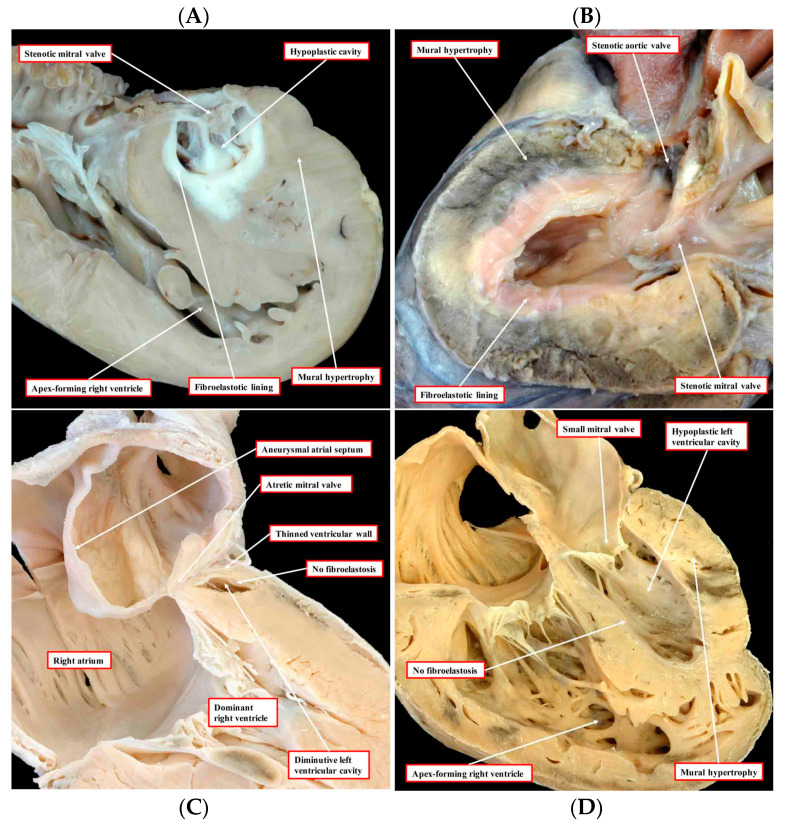
The images, all photographed by Diane E. Spicer and reproduced with her permission, show the phenotypic variants of hypoplastic left heart syndrome as seen in the clinical setting. The upper left panel (**A**) shows the variant with mitral stenosis and aortic atresia. The heart in the upper right-hand panel (**B**) has mitral and aortic stenosis. In the lower panels, to the left (**C**) is seen the variant with mitral atresia, and to the right (**D**) is the rarest variant with left ventricular hypoplasia with the small aortic and mitral valves, their size in keeping with that of the left ventricle, although the aortic valve is not seen in the four-chamber section through the heart [1].

**Figure 2:**
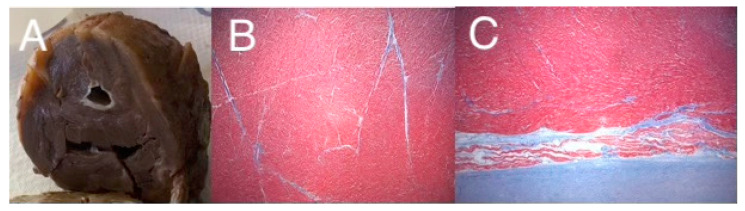
Gross specimen of an explanted heart from a patient with HLHS, with mitral stenosis and aortic stenosis (**A**), showing a diminutive LV chamber with endocardial fibroelastosis and a thickened LV wall, and trichrome staining from the LV free wall (**B**), and a luminal biopsy demonstrating severe EFE (**C**). Images graciously provided by Dr. Denise Malicki, Department of Pathology, Rady Children’s Hospital of San Diego.

“Classic” hypoplastic left heart syndrome is defined as hypoplasia of the LV chamber in association with stenosis and/or atresia of the mitral and aortic valves, hypoplasia of the aorta, and an intact interventricular septum [2]. As shown in Figure 1, there is significant variability among the HLHS anatomic subtypes. For example, in mitral and aortic atresia the left ventricle is typically “slit-like”, suggestive of decreased growth of all of the left-sided structures. In contrast, in the subset with aortic valve atresia with mitral valve stenosis, the left ventricular free wall is thickened, and there is frequently endocardial fibroelastosis. In an explanted HLHS heart with mitral stenosis and aortic stenosis and increased thickening of the LV free wall, we have observed an abundance of cardiac myocytes comprising the LV free wall (Figure 2). It is unknown how these anatomic subtypes are related mechanistically, specifically with respect to etiology, or at what stage during cardiac development the causal event(s) occurs.

Through combined human genetics and genetically-engineered animal model systems, we have identified the ETS1 transcription factor as the likely cause of congenital heart defects, including HLHS, in the rare chromosomal deletion disorder, Jacobsen syndrome (OMIM #147791) [3]. Remarkably, 5–10% of all infants born with JS have HLHS, one of the highest known frequencies of HLHS for any genetic syndrome. Mutations in ETS1 may account for about 1% of all cases of HLHS.

During murine heart development, ETS1 is only expressed in the cardiac neural crest and the endocardium/vascular endothelium but, interestingly, not in cardiac myocytes. Consequently, we have hypothesized that the loss of ETS1 in the neural crest and/or the endocardium/vascular endothelium can cause HLHS through a non-cell-autonomous effect on ventricular cardiac myocyte development and function.

## 2. A Role for ETS1 in Heart Cell Fate Determination and the “Hypoplastic” Paradox: Reconciling Animal Models with Human HLHS

**Figure 3:**
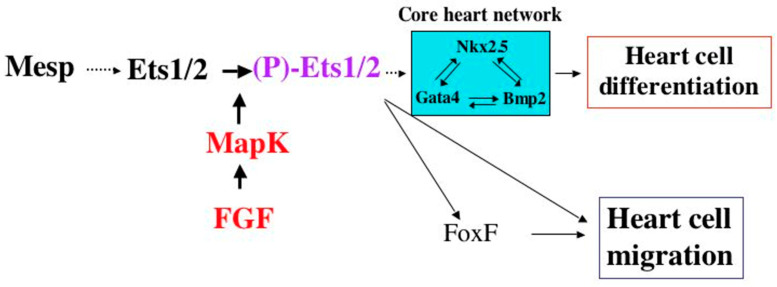
*Ciona* heart specification network. Schematic diagram depicting the transcriptional core network of genes involved in heart development in *Ciona intestinalis*. Mutations in at least three of these genes (ETS1, NKX2-5, and FoxF1) have been identified in patients in association with HLHS. (Kindly provided by Dr. Brad Davidson.)

Previous studies in the ascidian *Ciona intestinalis* have demonstrated a critical role for ETS1 in heart cell fate determination and cell migration, suggesting that the impairment of one or both of these two cellular functions might underlie the pathogenesis of HLHS (Figure 3) [4].

**Figure 4:**
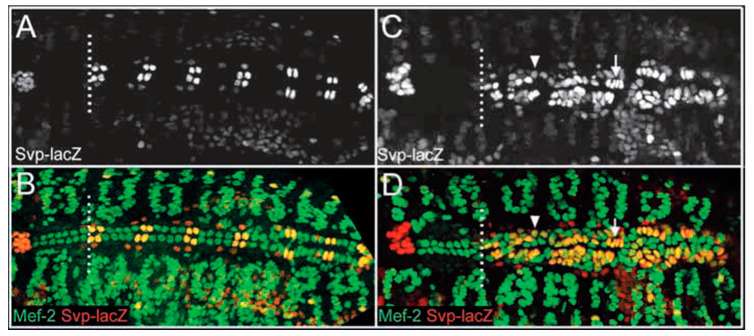
*Pointed* embryos exhibit a significant increase in Svp-positive cardioblasts. Dorsal views of stage 16 wild-type (**A**,**B**) and pnt (**C**,**D**) embryos labeled for Svp-lacZ (**A**,**C**), and Svp-lacZ and Mef-2 (**B**,**D**). (**A**,**B**) In wild-type embryos, 12 Svp-negative cardioblasts arise anterior to the first Svp-positive cardioblast (broken white line); seven of these cardioblasts are visible in A and B. Posterior to this location, there is a reiterative pattern of two Svp-lacZ positive cardioblasts (yellow/orange) and four Svp-lacZ negative cardioblasts (green) per hemisegment. (**C**,**D**) In pnt embryos, cardioblast development anterior to the first Svp-positive cardioblast (broken white line) appears normal. However, many ectopic cardioblasts are found posterior to this location and the majority of these cells express Svp-lacZ at high (arrow) or moderate levels (arrowhead). The broken white line separates the anterior heart domain from the posterior seven heart segments; anterior is towards the left [5].

Prior to the studies in *Ciona*, Alvarez et al. demonstrated that loss of function mutations in *Pointed* (*ETS1*) in *Drosophila* cause a loss of pericardial cells and a concomitant increase in the number of cardioblasts [5]. This process selectively involves the posterior aspect of the heart tube, which may be analogous to the left ventricle in a mammalian heart (Figure 4). They concluded that the abundance of cardioblasts was due to a cell fate switch in which *Pointed* promotes pericardial cell development and opposes cardioblast development. More recently, Nie and Bronner [6] demonstrated that knockdown of ETS1 in the cardiac mesoderm in *Xenopus* caused an HLHS-like ventricle characterized by an abundance of cardiac myocytes and a loss of endocardial cells, consistent with a role for ETS1 in regulating cell fate determination between endocardial cells and cardiac myocytes. Together, these results suggest that in these animal models, the HLHS-like ventricle may be due to a relative abundance of cardiac myocytes (and loss of endocardial cells) leading to a decreased ventricular chamber volume. An important issue that remains to be resolved is whether the total ventricular volume (i.e., including the walls) is decreased, and whether the absolute number of cardiac myocytes is increased in these animal models, i.e., paralleling the apparent increase in cardiac myocytes in the LV wall of the aortic atresia/mitral stenosis HLHS anatomic subtype. Interestingly, Bohlmeyer et al. [7] demonstrated the ectopic expression of CD31, a marker of endothelial cells, in cardiac myocytes in HLHS left ventricular tissue and postulated that this ectopic expression “could be an effect of upstream developmental program error that results in HLHS”, consistent with the observations in *Drosophila* and *Xenopus* for a role for ETS1 in regulating cell fate determination. Together, these studies provide evidence that a cell fate switch between endothelial/endocardium and cardiac myocytes may be a critical component in the pathogenesis of HLHS in such patients. In addition, Miao et al., reported in vitro studies demonstrating impaired endocardial function in cells derived from patients with HLHS, coinciding with decreased ETS1 expression [8]. Contrary to previous studies [2], this implies that for this subset of patients, the molecular and cellular events underlying HLHS occur very early in cardiac development.

Interestingly, in most cases of HLHS in patients with Jacobsen syndrome, the HLHS anatomic subtype is aortic and mitral valve atresia with a slit-like, diminutive left ventricle (Grossfeld P, unpublished results). This anatomic subtype is suggestive of a growth arrest, and previously published studies on cardiac myocytes derived from iPS cells from patients with HLHS indicate decreased cardiac myocyte proliferation [9,10,11,12,13], as well as in co-culture studies reported by Maio et al. [8]. Notably, Snider et al. [14] demonstrated that early genetic ablation of endocardial cells in mice leads to a severely hypoplastic, growth-arrested heart that arguably resembles the “slit-like” LV in the AA/MA subtype. These studies provide further support for a critical role for the endocardium in the pathogenesis of HLHS in at least a subset of patients, and suggest that in patients with JS and HLHS, it is the early loss of ETS1 and endocardial function that leads to irreversible growth arrest of the left-sided structures.

**Figure 5:**
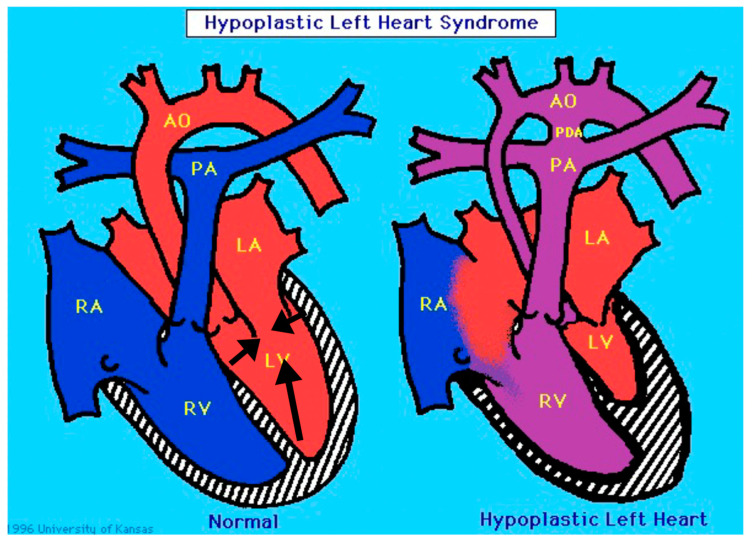
Schematic diagram model for one potential mechanism for the aortic atresia/mitral stenosis anatomic subtype of HLHS, indicating “concentric hyperplasia”, i.e., inward growth of cardiac myocytes leading to a thickened ventricular wall and diminutive chamber volume.

The notion of increased cardiac myocyte numbers in the HLHS LV would seem to contradict those reports of decreased proliferation in cardiac myocytes derived from HLHS patients. One possible explanation is that an increased cardiac myocyte number could be due to a loss of ETS1/endocardial function at a later stage of cardiac development, in which the diminutive chamber volume results directly from the excessive proliferation of cardiac myocytes, as opposed to an arrest of growth. This would imply the presence of one or more factors that drive increased cardiac myocyte proliferation in the HLHS left ventricle. In that case, early growth arrest would result from the absence of such a factor(s), whereas causal events occurring later in development would not inhibit such a factor(s), thereby promoting increased cardiac myocyte proliferation. Interestingly, it has been observed that in some cases of critical aortic valve stenosis in the fetus, the left ventricle is initially dilated and poorly functioning, with subsequent evolution toward a thick-walled “hypoplastic” left ventricle. This would imply a process in which there is an inward growth of cardiac myocytes, i.e., “concentric hyperplasia” (Figure 5). The molecular and cellular mechanisms underlying that transition are completely unknown. Lastly, it is unknown whether cardiac myocytes derived from a patient with the AA/MS anatomic subtype of HLHS may also exhibit decreased proliferation or in fact could have increased proliferative capacity.

## 3. The Cardiac Myocyte and HLHS: Clinical Implications

As discussed above, our studies demonstrate that ETS1 is not expressed in cardiac myocytes during murine heart development. Although most studies to date have demonstrated defects in cardiac myocytes derived from HLHS patients, the absence of expression of ETS1 in cardiac myocytes would suggest that these cardiac myocytes are intrinsically normal. Consistent with this, previous studies in chick [15,16] and more recently in mice [17] prove that, at least in these models of HLHS in which inflow across the mitral valve into the developing left ventricle is restricted, the cardiac myocytes from the “HLHS” LV are genetically normal. Together these results suggest there is a subset of HLHS patients in which the LV cardiac myocytes might have normal growth potential and function.

## 4. Future Directions

Our studies in mice and previous studies in *Xenopus* and in endothelial cells derived from iPS cells from HLHS patients all point to a critical role for the endocardium in the pathogenesis of HLHS. Future studies will focus on identifying the signaling pathways between endocardium and ventricular myocytes that are perturbed in HLHS. Such studies may help to identify novel therapeutic targets that could potentially restore endocardial function and prevent the development of HLHS. In retrospect, while attempts to intervene by fetal balloon angioplasty to relieve critical aortic stenosis are noble [18], the overall low success rate of this procedure suggests that for the majority of patients, it is performed beyond the developmental window of opportunity in which normal LV growth can proceed or be restored. This would be consistent with a critical role for the endocardium in the pathogenesis of HLHS in a large subset of patients. Notwithstanding this, there may be select patients with intrinsically normal cardiac myocytes that may respond to fetal balloon angioplasty and potentially future therapies aimed at salvaging the HLHS left ventricle, thereby allowing for a two-ventricle approach (i.e., in cases where the defect occurs late in cardiac development). As alluded to above, based on the phenotypic heterogeneity, there are likely to be multiple mechanisms that underlie the pathogenesis of HLHS. One important area of future research will be to characterize further cardiac myocytes from HLHS left ventricles, to determine if these cells represent a unique population of cardiac myocytes, as suggested from Bohlmeyer’s studies, or in other cases are intrinsically normal.

**Figure 6:**
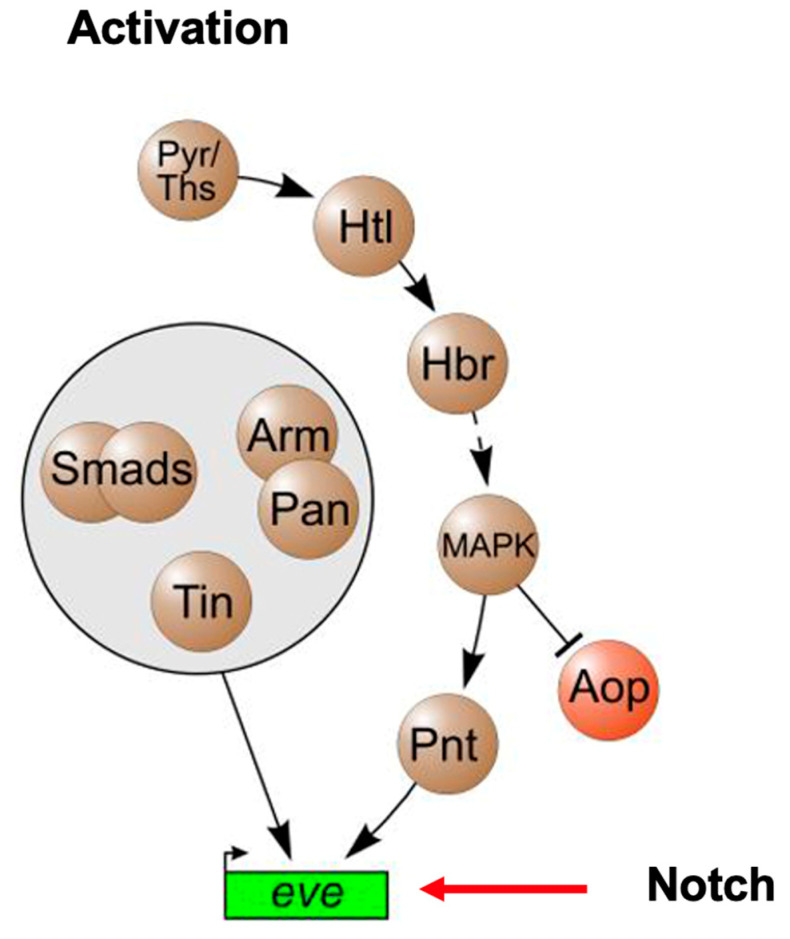
Cardiac mesoderm activation pathways in *Drosophila* heart development (adapted from Bryantsev and Cripps, 2009, Biochim Biophys Acta. 2009 April; 1789(4): 343–353) [19].

Early studies in *Drosophila* have led to a remarkable observation that there is a conserved network of cardiac transcription factors required for early heart development that includes *Notch*, *Tinman* (*NKX2-5*), and *Pointed* (*ETS1*) [19] (Figure 6). Mutations in all three of these genes have been identified in HLHS patients, suggesting that this “HLHS core transcriptional network” has been conserved through evolution, and that these HLHS disease-causing genes play a fundamental and critical role in heart development across species. Similarly, *NKX2-5* and *ETS1* as well as another HLHS-associated gene, *FOXF1*, comprise the cardiac transcriptional core network of genes in *Ciona* heart development. Interestingly, in *Drosophila,* while the loss of function mutations in *Tinman* result in the complete absence of a developing heart, the loss of function mutations in *Notch* cause the same cardiac phenotype observed in *Pointed* mutant hearts, i.e., a loss of pericardial cells and an increase in cardiomyocytes leading to an obliterated ventricular lumen [20]. It will be exciting to learn what additional downstream factors the “HLHS network” involving these genes regulate, and if they too are involved in the pathogenesis of HLHS.

## Data Availability

Not applicable.

## References

[1] Grossfeld P., Nie S., Lin L., Wang L., Anderson R.H. (2019). Hypoplastic Left Heart Syndrome: A New Paradigm for an Old Disease?. J. Cardiovasc. Dev. Dis..

[2] Crucean A., Alqahtani A., Barron D.J., Brawn W.J., Richardson R.V., O’Sullivan J., Anderson R.H., Henderson D.J., Chaudhry B. (2017). Re-evaluation of hypoplastic left heart syndrome from a developmental and morphological perspective. Orphanet J. Rare Dis..

[3] Ye M., Coldren C., Liang X., Mattina T., Goldmuntz E., Benson D.W., Ivy D., Perryman M.B., Garrett-Sinha L.A., Grossfeld P. (2010). Deletion of ETS-1, a gene in the Jacobsen syndrome critical region, causes ventricular septal defects and abnormal ventricular morphology in mice. Hum. Mol. Genet..

[4] Davidson B., Shi W., Levine M. (2005). Uncoupling heart cell specification and migration in the simple chordate Ciona intestinalis. Development.

[5] Alvarez A.D., Shi W., Wilson B.A., Skeath J.B. (2003). pannier and pointedP2 act sequentially to regulate Drosophila heart development. Development.

[6] Nie S., Bronner M.E. (2015). Dual developmental role of transcriptional regulator Ets1 in Xenopus cardiac neural crest vs. heart mesoderm. Cardiovasc. Res..

[7] Bohlmeyer T.J., Helmke S., Ge S., Lynch J., Brodsky G., Sederberg J.H., Robertson A.D., Minobe W., Bristow M.R., Perryman M.B. (2003). Hypoplastic left heart syndrome myocytes are differentiated but possess a unique phenotype. Cardiovasc. Pathol..

[8] Miao Y., Tian L., Martin M., Paige S.L., Galdos F.X., Li J., Klein A., Zhang H., Ma N., Wei Y. (2020). Intrinsic Endocardial Defects Contribute to Hypoplastic Left Heart Syndrome. Cell Stem Cell.

[9] Paige S.L., Galdos F.X., Lee S., Chin E.T., Ranjbarvaziri S., Feyen D.A.M., Darsha A.K., Xu S., Ryan J.A., Beck A.L. (2020). Patient-Specific Induced Pluripotent Stem Cells Implicate Intrinsic Impaired Contractility in Hypoplastic Left Heart Syndrome. Circulation.

[10] Kobayashi J., Yoshida M., Tarui S., Hirata M., Nagai Y., Kasahara S., Naruse K., Ito H., Sano S., Oh H. (2014). Directed differentiation of patient-specific induced pluripotent stem cells identifies the transcriptional repression and epigenetic modification of NKX2-5, HAND1, and NOTCH1 in hypoplastic left heart syndrome. PLoS ONE.

[11] Jiang Y., Habibollah S., Tilgner K., Collin J., Barta T., Al-Aama J.Y., Tesarov L., Hussain R., Trafford A.W., Kirkwood G. (2014). An induced pluripotent stem cell model of hypoplastic left heart syndrome (HLHS) reveals multiple expression and functional differences in HLHS-derived cardiac myocytes. Stem Cells Transl. Med..

[12] Hrstka S.C., Li X., Nelson T.J., Wanek Program Genetics Pipeline G. (2017). NOTCH1-Dependent Nitric Oxide Signaling Deficiency in Hypoplastic Left Heart Syndrome Revealed Through Patient-Specific Phenotypes Detected in Bioengineered Cardiogenesis. Stem Cells.

[13] Theis J.L., Hrstka S.C., Evans J.M., O’Byrne M.M., de Andrade M., O’Leary P.W., Nelson T.J., Olson T.M. (2015). Compound heterozygous NOTCH1 mutations underlie impaired cardiogenesis in a patient with hypoplastic left heart syndrome. Hum. Genet..

[14] Snider P., Simmons O., Wang J., Hoang C.Q., Conway S.J. (2014). Ectopic Noggin in a Population of Nfatc1 Lineage Endocardial Progenitors Induces Embryonic Lethality. J. Cardiovasc. Dev. Dis..

[15] deAlmeida A., McQuinn T., Sedmera D. (2007). Increased ventricular preload is compensated by myocyte proliferation in normal and hypoplastic fetal chick left ventricle. Circ. Res..

[16] Pesevski Z., Kvasilova A., Stopkova T., Nanka O., Drobna Krejci E., Buffinton C., Kockova R., Eckhardt A., Sedmera D. (2018). Endocardial Fibroelastosis is Secondary to Hemodynamic Alterations in the Chick Embryonic Model of Hypoplastic Left Heart Syndrome. Dev. Dyn..

[17] Rahman A., DeYoung T., Cahill L.S., Yee Y., Debebe S.K., Botelho O., Seed M., Chaturvedi R.R., Sled J.G. (2021). A mouse model of hypoplastic left heart syndrome demonstrating left heart hypoplasia and retrograde aortic arch flow. Dis. Models Mech..

[18] Tworetzky W., Wilkins-Haug L., Jennings R.W., van der Velde M.E., Marshall A.C., Marx G.R., Colan S.D., Benson C.B., Lock J.E., Perry S.B. (2004). Balloon dilation of severe aortic stenosis in the fetus: Potential for prevention of hypoplastic left heart syndrome: Candidate selection, technique, and results of successful intervention. Circulation.

[19] Bryantsev A., Cripps R.M. (2009). Cardiac gene regulatory networks in Drosophila. Biochim. Biophys. Acta.

[20] Hartenstein A.Y., Rugendorff A., Tepass U., Hartenstein V. (1992). The function of the neurogenic genes during epithelial development in the Drosophila embryo. Development.

